# Supplementary material for: Changes of Volatile Organic Compounds of Different Flesh Texture Pears during Shelf Life Based on Headspace Solid-Phase Microextraction with Gas Chromatography–Mass Spectrometry
Source: Foods. 2023 Nov 23;12(23):4224. doi: 10.3390/foods12234224 (PMC10706164; doi:10.3390/foods12234224)
Supplement: Supplementary file 1 [file foods-12-04224-s001.zip › foods-2706136-supplementary.pdf]

Supplementary Materials

Table S1. VOCs content, VIP value and *P* value of eight pear varieties at different shelf life

Code	Volatile substance name	CAS	Content of VOCs in the flesh of Korla pear (mg/kg FW)					Content of VOCs in the flesh of Yali (mg/kg FW)				
			0 d	3 d	7 d	14 d	21 d	0 d	3 d	7 d	14 d	21 d
3	DL-2-Methylbutanol	1565-80-6	—	—	—	—	—	—	—	—	—	—
4	1-Hexanol	111-27-3	0.51±0.15	1.00±0.22	0.80±0.32	1.03±0.17	0.55±0.09	1.28±0.18	1.08±0.09	0.79±0.03	0.72±0.07	0.48±0.06
6	trans-3-Hexenol	928-97-2	0.08±0.02	0.06±0.01	0.02±0.01	—	—	0.23±0.03	0.12±0.01	0.03±0.00	—	—
8	(E)-2-Hexen-1-ol	928-95-0	0.04±0.01	0.04±0.01	0.02±0.01	0.03±0.01	0.02±0.00	0.15±0.02	0.10±0.01	0.11±0.00	0.08±0.00	0.04±0.00
9	1-Octen-3-ol	3391-86-4	0.22±0.03	0.14±0.00	0.05±0.00	—	—	0.09±0.01	0.23±0.02	0.17±0.01	—	—
13	Linalool	78-70-6	—	—	—	—	—	0.06±0.01	0.07±0.01	0.07±0.01	0.07±0.01	0.06±0.01
22	α-Terpineol	98-55-5	—	—	—	—	—	0.05±0.01	0.10±0.01	0.08±0.00	0.09±0.01	0.07±0.01
45	Methyl butanoate	623-42-7	—	—	—	—	—	—	—	—	—	—
48	Ethyl butanoate	105-54-4	—	—	—	—	0.05±0.01	—	—	0.03±0.00	0.50±0.21	1.14±0.08
49	Ethyl 2-methylbutanoate	7452-79-1	—	—	—	—	—	—	—	—	0.03±0.01	0.11±0.01
50	Butyl acetate	123-86-4	—	—	—	0.02±0.00	0.03±0.01	0.02±0.00	0.02±0.00	0.02±0.00	0.03±0.01	0.03±0.00
57	Methyl caproate	106-70-7	—	—	—	—	—	—	—	—	—	—
61	Ethyl caproate	123-66-0	—	—	—	0.01±0.00	0.05±0.02	—	—	—	0.40±0.16	0.94±0.03
64	Hexyl acetate	142-92-7	0.10±0.03	0.16±0.07	0.06±0.03	0.14±0.02	0.16±0.05	0.35±0.13	0.58±0.05	0.23±0.03	0.25±0.12	0.38±0.01
67	(E)-3-Hexenol acetate	3681-82-1	0.07±0.00	0.07±0.02	—	—	—	0.13±0.02	0.21±0.02	0.02±0.00	—	—
79	Ethyl (E)-2-octenoate	7367-82-0	—	—	—	—	—	—	—	—	—	—
85	Ethyl 3-hydroxyhexanoate	2305-25-1	—	—	—	—	—	—	—	—	0.07±0.01	0.21±0.04
92	Phenethyl acetate	103-45-7	—	—	—	0.03±0.00	0.02±0.00	—	—	—	—	—
93	(Z,E)-methyl-2,4-decadienoate	108965-84-0	—	—	—	—	—	—	—	—	—	—
94	Ethyl (E,Z)-2,4-decadienoate	3025-30-7	—	—	—	—	—	—	—	—	—	—
102	Hexanal	66-25-1	2.42±0.21	2.15±0.29	2.20±0.46	3.85±0.12	3.29±0.41	1.27±0.18	1.19±0.08	1.38±0.14	1.03±0.34	1.14±0.04
104	(E)-2-Hexanal	6728-26-3	0.65±0.14	0.81±0.12	0.85±0.10	1.20±0.04	0.85±0.08	0.59±0.14	0.45±0.04	0.71±0.07	0.39±0.07	0.31±0.03
106	(E)-2-Heptenal	18829-55-5	0.62±0.09	0.32±0.02	0.06±0.01	0.08±0.02	0.18±0.02	0.08±0.02	0.38±0.02	0.16±0.02	0.13±0.02	—
108	(E)-2-Octenal	2548-87-0	0.47±0.11	0.24±0.02	0.08±0.01	0.14±0.01	0.20±0.03	0.06±0.00	0.26±0.02	0.16±0.02	0.17±0.03	0.10±0.01
113	(E)-2-Decenal	3913-81-3	0.03±0.01	—	—	—	—	—	0.02±0.00	0.01±0.00	0.01±0.00	—
125	1-Hepten-3-one	2918-13-0	0.16±0.02	0.09±0.00	0.03±0.01	0.03±0.00	0.03±0.00	0.03±0.01	0.13±0.01	0.09±0.01	0.06±0.02	0.07±0.01
126	6-Methyl-5-heptene-2-one	110-93-0	0.05±0.01	0.03±0.00	0.02±0.00	0.02±0.00	0.01±0.00	0.04±0.01	—	0.02±0.00	0.03±0.00	0.02±0.00
128	(E)-1-(2,6,6-Trimethyl-1,3-cyclohexadien-1-yl)-2-buten-1-one	23726-93-4	—	—	—	0.01±0.00	—	0.02±0.01	0.05±0.01	0.04±0.00	0.06±0.01	0.06±0.01
148	(Z,E)-α-Farnesene	26560-14-5	—	—	—	—	—	—	—	—	—	—
149	(E,E)-α-Farnesene	502-61-4	—	—	—	—	—	—	—	—	—	—
155	trans-α,α,5-Trimethyl-5-ethenyltetrahydro-2-furanmethanol	34995-77-2	0.08±0.01	0.08±0.00	0.06±0.01	0.09±0.01	0.05±0.01	0.25±0.03	0.33±0.03	0.22±0.00	0.23±0.01	0.18±0.03
159	3-Allyl-6-methoxyphenol	501-19-9	—	—	—	—	—	0.18±0.02	0.17±0.01	—	—	—

Table S1 (*continued*)

Code	Volatile substance name	CAS	Content of VOCs in the flesh of Hanhongli (mg/kg FW)					Content of VOCs in the flesh of Nanguoli (mg/kg FW)				
			0 d	3 d	7 d	14 d	21 d	0 d	3 d	7 d	14 d	21 d
3	DL-2-Methylbutanol	1565-80-6	—	—	—	—	—	—	—	—	—	—
4	1-Hexanol	111-27-3	0.68±0.05	1.01±0.09	0.99±0.20	0.90±0.10	0.66±0.09	0.88±0.16	1.25±0.25	0.98±0.12	0.63±0.15	0.90±0.24
6	trans-3-Hexenol	928-97-2	0.49±0.03	0.31±0.03	0.19±0.02	—	—	0.14±0.02	0.03±0.01	0.01±0.00	—	—
8	(E)-2-Hexen-1-ol	928-95-0	0.02±0.00	0.07±0.02		0.08±0.01	0.06±0.01	0.09±0.02	0.15±0.04	0.10±0.01	0.07±0.03	0.06±0.01
9	1-Octen-3-ol	3391-86-4	—	—	—	—	—	—	—	—	—	—
13	Linalool	78-70-6	—	—	—	—	—	—	—	—	—	—
22	α-Terpineol	98-55-5	—	—	—	—	—	—	—	—	—	—
45	Methyl butanoate	623-42-7	—	—	—	0.05±0.02	0.02±0.00	—	0.01±0.00	—	0.05±0.01	0.11±0.08
48	Ethyl butanoate	105-54-4	—	0.01±0.00	0.01±0.00	0.92±0.11	0.54±0.05	0.11±0.02	0.70±0.08	0.41±0.11	1.65±0.11	2.42±1.70
49	Ethyl 2-methylbutanoate	7452-79-1	—	—	—	0.03±0.00	0.04±0.00	0.05±0.01	0.16±0.02	0.11±0.03	0.40±0.04	0.95±0.78
50	Butyl acetate	123-86-4	0.01±0.00	0.01±0.00	—	0.05±0.01	0.03±0.00	0.19±0.06	0.38±0.11	0.12±0.03	0.09±0.01	0.14±0.09
57	Methyl caproate	106-70-7	—	—	—	0.13±0.03	0.06±0.00	—	0.05±0.02	0.02±0.01	0.26±0.01	0.45±0.35
61	Ethyl caproate	123-66-0	—	—	—	1.90±0.19	1.03±0.07	0.08±0.01	2.77±0.24	1.15±0.42	8.29±0.22	9.73±5.75
64	Hexyl acetate	142-92-7	0.09±0.01	0.43±0.10	0.27±0.07	0.54±0.06	0.30±0.07	0.58±0.03	4.24±0.47	1.29±0.41	2.24±0.01	3.09±1.95
67	(E)-3-Hexenol acetate	3681-82-1	0.31±0.03	0.49±0.06	0.21±0.06	—	—	0.09±0.05	—	—	—	—
79	Ethyl (E)-2-octenoate	7367-82-0	—	—	—	0.06±0.01	—	—	—	—	0.62±0.05	0.69±0.38
85	Ethyl 3-hydroxyhexanoate	2305-25-1	—	—	—	0.32±0.05	0.39±0.06	—	0.02±0.00	—	0.49±0.04	0.99±0.23
92	Phenethyl acetate	103-45-7	—	—	—	0.05±0.02	0.03±0.00	—	0.37±0.05	—	0.23±0.02	0.08±0.04
93	(Z,E)-methyl-2,4-decadienoate	108965-84-0	—	—	—	—	—	—	—	—	0.11±0.01	0.33±0.21
94	Ethyl (E,Z)-2,4-decadienoate	3025-30-7	—	—	—	0.04±0.01	0.02±0.00	—	0.02±0.00	—	1.13±0.09	2.49±1.42
102	Hexanal	66-25-1	1.12±0.20	0.72±0.18	0.82±0.21	0.99±0.33	0.79±0.08	1.34±0.50	0.81±0.22	0.87±0.16	0.55±0.14	0.63±0.39
104	(E)-2-Hexanal	6728-26-3	0.25±0.07	0.66±0.04	0.80±0.12	0.58±0.11	0.57±0.11	1.32±0.54	1.63±0.33	1.43±0.30	0.84±0.10	0.71±0.25
106	(E)-2-Heptenal	18829-55-5	—	0.11±0.05	0.07±0.02	0.14±0.01	0.04±0.00	0.03±0.00	—	0.05±0.01	0.04±0.02	—
108	(E)-2-Octenal	2548-87-0	0.03±0.01	0.10±0.01	0.09±0.01	0.20±0.03	0.07±0.00	0.03±0.01	0.09±0.02	0.06±0.01	0.08±0.00	0.08±0.05
113	(E)-2-Decenal	3913-81-3	—	—	—	—	—	—	—	—	—	—
125	1-Hepten-3-one	2918-13-0	—	0.05±0.03	0.02±0.01	—	—	0.01±0.00	—	—	—	—
126	6-Methyl-5-heptene-2-one	110-93-0	—	0.02±0.01	—	0.02±0.00	—	—	0.03±0.00	0.02±0.00	0.12±0.01	0.12±0.06
128	(E)-1-(2,6,6-Trimethyl-1,3-cyclohexadien-1-yl)-2-buten-1-one	23726-93-4	0.13±0.03	0.27±0.06	0.28±0.09	0.42±0.13	0.31±0.07	0.11±0.05	0.22±0.10	0.21±0.06	0.20±0.05	0.16±0.08
148	(Z,E)-α-Farnesene	26560-14-5	—	—	—	—	—	—	—	—	0.18±0.03	0.16±0.09
149	(E,E)-α-Farnesene	502-61-4	—	—	—	—	—	—	0.15±0.04	0.06±0.02	4.86±0.90	4.63±2.48
155	trans-α,α,5-Trimethyl-5-ethenyltetrahydro-2-furanmethanol	34995-77-2	0.29±0.01	0.22±0.01	0.29±0.07	0.24±0.05	0.16±0.03	0.32±0.09	0.60±0.20	0.56±0.07	0.34±0.10	0.29±0.08
159	3-Allyl-6-methoxyphenol	501-19-9	—	—	—	—	—	—	—	—	—	—

Table S1 (*continued*)

Code	Volatile substance name	CAS	Content of VOCs in the flesh of Jingbaili (mg/kg FW)					Content of VOCs in the flesh of Ruanerli (mg/kg FW)				
			0 d	3 d	7 d	14 d	21 d	0 d	3 d	7 d	14 d	21 d
3	DL-2-Methylbutanol	1565-80-6	—	—	—	—	—	—	—	—	—	—
4	1-Hexanol	111-27-3	1.05±0.15	1.10±0.17	1.03±0.06	0.90±0.02	1.01±0.14	1.41±0.20	1.55±0.08	1.23±0.14	1.08±0.11	0.53±0.03
6	trans-3-Hexenol	928-97-2	0.55±0.06	0.07±0.01	—	—	—	—	—	—	—	—
8	(E)-2-Hexen-1-ol	928-95-0	0.01±0.00	0.14±0.01	0.14±0.01	0.09±0.01	0.06±0.01	0.19±0.03	0.12±0.01	0.04±0.01	0.06±0.00	0.03±0.00
9	1-Octen-3-ol	3391-86-4	0.10±0.04	—	—	—	—	—	—	—	—	—
13	Linalool	78-70-6	—	—	0.01±0.00	0.07±0.00	0.02±0.00	0.08±0.01	0.04±0.00	0.03±0.00	0.07±0.01	0.05±0.00
22	α-Terpineol	98-55-5	—	—	—	0.05±0.00	—	0.42±0.03	0.47±0.04	0.31±0.04	0.52±0.08	0.36±0.02
45	Methyl butanoate	623-42-7	—	0.01±0.00	0.06±0.01	0.68±0.04	0.50±0.04	0.06±0.02	0.12±0.03	0.14±0.02	0.11±0.04	0.02±0.00
48	Ethyl butanoate	105-54-4	—	—	—	0.04±0.00	0.07±0.01	0.43±0.08	0.92±0.06	0.65±0.02	0.43±0.08	0.18±0.01
49	Ethyl 2-methylbutanoate	7452-79-1	—	—	—	—	0.02±0.00	0.02±0.00	0.04±0.00	0.09±0.01	0.08±0.01	0.09±0.01
50	Butyl acetate	123-86-4	—	—	0.03±0.00	0.09±0.00	0.07±0.00	1.56±0.34	1.14±0.21	0.94±0.06	0.29±0.06	0.11±0.01
57	Methyl caproate	106-70-7	—	—	0.08±0.01	0.91±0.13	0.82±0.08	0.20±0.07	0.58±0.04	0.71±0.03	0.57±0.10	0.15±0.01
61	Ethyl caproate	123-66-0	—	—	—	0.06±0.01	0.23±0.03	0.56±0.20	3.54±0.03	3.30±0.28	2.35±0.42	1.05±0.07
64	Hexyl acetate	142-92-7	0.40±0.22	0.75±0.12	1.25±0.26	1.93±0.13	1.75±0.15	1.58±0.49	6.33±0.47	5.82±0.20	2.11±0.36	0.74±0.05
67	(E)-3-Hexenol acetate	3681-82-1	0.52±0.10	0.10±0.01	—	—	—	—	—	—	—	—
79	Ethyl (E)-2-octenoate	7367-82-0	—	—	—	—	—	—	—	—	—	—
85	Ethyl 3-hydroxyhexanoate	2305-25-1	—	—	—	—	0.02±0.00	—	0.06±0.00	0.07±0.01	0.09±0.01	0.11±0.00
92	Phenethyl acetate	103-45-7	—	—	0.01±0.00	0.03±0.00	0.07±0.02	0.03±0.00	0.25±0.02	0.27±0.02	0.62±0.13	0.09±0.01
93	(Z,E)-methyl-2,4-decadienoate	108965-84-0	—	—	—	0.09±0.01	0.21±0.04	—	—	—	—	—
94	Ethyl (E,Z)-2,4-decadienoate	3025-30-7	—	—	—	—	—	—	—	—	—	0.03±0.00
102	Hexanal	66-25-1	2.45±0.30	2.24±0.20	1.92±0.06	1.78±0.14	2.28±0.25	2.36±0.27	1.52±0.08	1.61±0.04	2.27±0.30	1.10±0.03
104	(E)-2-Hexanal	6728-26-3	1.34±0.15	1.95±0.23	1.68±0.26	0.95±0.02	0.63±0.04	2.55±0.40	1.83±0.13	1.39±0.06	1.25±0.11	0.52±0.07
106	(E)-2-Heptenal	18829-55-5	0.13±0.04	0.30±0.02	0.10±0.01	0.09±0.01	0.08±0.01	—	0.08±0.01	0.06±0.01	0.08±0.01	—
108	(E)-2-Octenal	2548-87-0	0.09±0.02	0.23±0.02	0.15±0.01	0.14±0.01	0.13±0.02	0.05±0.01	0.16±0.00	0.14±0.01	0.14±0.01	0.13±0.02
113	(E)-2-Decenal	3913-81-3	—	—	—	—	—	—	—	—	—	—
125	1-Hepten-3-one	2918-13-0	0.05±0.02	0.06±0.01	0.02±0.00	—	0.02±0.00	—	0.02±0.00	—	0.03±0.01	0.04±0.01
126	6-Methyl-5-heptene-2-one	110-93-0	—	0.04±0.00	0.08±0.00	0.25±0.01	0.10±0.01	—	0.02±0.00	0.02±0.00	0.01±0.00	0.01±0.00
128	(E)-1-(2,6,6-Trimethyl-1,3-cyclohexadien-1-yl)-2-buten-1-one	23726-93-4	0.10±0.02	0.15±0.03	0.20±0.02	0.23±0.01	0.21±0.04	0.55±0.10	0.38±0.10	0.28±0.07	0.51±0.14	0.33±0.05
148	(Z,E)-α-Farnesene	26560-14-5	—	—	0.05±0.01	0.39±0.07	0.19±0.04	—	—	—	—	—
149	(E,E)-α-Farnesene	502-61-4	—	0.04±0.00	0.52±0.06	3.76±0.50	1.50±0.24	—	—	—	—	—
155	trans-α,α,5-Trimethyl-5-ethenyltetrahydro-2-furanmethanol	34995-77-2	0.22±0.01	0.27±0.03	0.28±0.03	0.24±0.02	0.22±0.02	2.87±0.14	1.75±0.12	1.61±0.32	1.21±0.10	0.90±0.04
159	3-Allyl-6-methoxyphenol	501-19-9	—	—	—	—	—	—	—	—	—	—

Table S1 (*continued*)

Code	Volatile substance name	CAS	Content of VOCs in the flesh of Cure (mg/kg FW)					Content of VOCs in the flesh of Louis (mg/kg FW)					VIP value	*P* value
			0 d	3 d	7 d	14 d	21 d	0 d	3 d	7 d	14 d	21 d		
3	DL-2-Methylbutanol	1565-80-6	—	—	0.02±0.00	0.01±0.00	—	—	—	—	—	0.54±0.07	1.44	0.00
4	1-Hexanol	111-27-3	0.44±0.02	0.37±0.03	0.36±0.06	0.20±0.02	0.16±0.02	0.23±0.02	0.40±0.03	0.27±0.04	0.15±0.02	0.52±0.07	2.10	0.00
6	trans-3-Hexenol	928-97-2	0.26±0.01	0.21±0.01	0.15±0.03	0.11±0.00	0.06±0.00	0.19±0.02	0.22±0.01	0.13±0.02	0.06±0.01	—	1.49	0.00
8	(E)-2-Hexen-1-ol	928-95-0	—	—	—	—	—	0.02±0.01	—	—	—	—	1.01	0.00
9	1-Octen-3-ol	3391-86-4	0.34±0.05	0.35±0.04	0.22±0.02	0.16±0.01	0.15±0.04	0.16±0.03	0.28±0.06	0.19±0.02	0.31±0.04	—	1.33	0.00
13	Linalool	78-70-6	0.01±0.00	0.01±0.00	—	—	—	0.01±0.00	—	—	—	0.02±0.00	1.10	0.00
22	α-Terpineol	98-55-5	—	0.01±0.00	—	—	—	—	—	—	—	0.04±0.00	1.46	0.00
45	Methyl butanoate	623-42-7	—	—	—	—	—	—	—	—	—	—	1.42	0.00
48	Ethyl butanoate	105-54-4	—	—	—	—	—	—	—	—	—	—	2.27	0.00
49	Ethyl 2-methylbutanoate	7452-79-1	—	—	—	—	—	—	—	—	—	—	1.27	0.00
50	Butyl acetate	123-86-4	—	—	—	—	—	—	—	—	—	0.15±0.02	1.89	0.00
57	Methyl caproate	106-70-7	—	—	—	—	—	—	—	—	—	—	1.69	0.00
61	Ethyl caproate	123-66-0	—	—	—	—	—	—	—	—	—	—	4.40	0.00
64	Hexyl acetate	142-92-7	—	—	—	—	—	—	—	—	—	0.19±0.04	3.67	0.00
67	(E)-3-Hexenol acetate	3681-82-1	—	—	—	—	—	—	—	—	—	—	1.75	0.00
79	Ethyl (E)-2-octenoate	7367-82-0	—	—	—	—	—	—	—	—	—	—	1.16	0.02
85	Ethyl 3-hydroxyhexanoate	2305-25-1	—	—	—	—	—	—	—	—	—	—	1.30	0.00
92	Phenethyl acetate	103-45-7	—	—	—	—	—	—	—	—	—	—	1.01	0.00
93	(Z,E)-methyl-2,4-decadienoate	108965-84-0	—	—	—	—	—	—	—	—	—	0.68±0.13	1.57	0.00
94	Ethyl (E,Z)-2,4-decadienoate	3025-30-7	—	—	—	—	—	—	—	—	—	1.25±0.21	2.21	0.00
102	Hexanal	66-25-1	0.86±0.03	0.38±0.03	0.47±0.12	0.44±0.01	0.42±0.00	0.34±0.07	0.34±0.01	0.25±0.03	0.28±0.02	0.42±0.06	3.91	0.00
104	(E)-2-Hexanal	6728-26-3	0.34±0.13	0.39±0.02	0.33±0.08	0.21±0.04	0.09±0.02	0.18±0.06	0.37±0.05	0.27±0.04	0.31±0.04	0.58±0.09	2.38	0.00
106	(E)-2-Heptenal	18829-55-5	0.95±0.13	1.04±0.15	0.57±0.04	0.40±0.01	0.40±0.15	0.42±0.07	0.97±0.24	0.56±0.11	1.11±0.13	0.38±0.04	2.14	0.00
108	(E)-2-Octenal	2548-87-0	0.30±0.05	0.44±0.10	0.27±0.01	0.21±0.01	0.32±0.13	0.12±0.02	0.51±0.10	0.28±0.05	0.63±0.05	0.22±0.02	1.45	0.00
113	(E)-2-Decenal	3913-81-3	0.09±0.02	0.09±0.02	0.04±0.00	0.04±0.01	0.07±0.04	0.04±0.01	0.18±0.04	0.09±0.02	0.35±0.03	0.04±0.00	1.04	0.00
125	1-Hepten-3-one	2918-13-0	0.48±0.09	0.40±0.09	0.18±0.02	0.13±0.01	0.20±0.09	0.11±0.03	0.31±0.09	0.22±0.05	0.53±0.07	0.12±0.02	1.39	0.00
126	6-Methyl-5-heptene-2-one	110-93-0	0.30±0.06	0.24±0.05	0.21±0.03	0.14±0.00	0.12±0.02	0.12±0.02	0.17±0.01	0.13±0.01	0.12±0.00	0.10±0.00	1.43	0.00
128	(E)-1-(2,6,6-Trimethyl-1,3-cyclohexadien-1-yl)-2-buten-1-one	23726-93-4	0.33±0.05	0.40±0.13	0.53±0.30	0.49±0.09	0.48±0.12	0.15±0.05	0.37±0.03	0.27±0.03	0.40±0.12	0.32±0.07	2.16	0.00
148	(Z,E)-α-Farnesene	26560-14-5	—	—	—	—	—	—	—	—	—	0.04±0.01	1.05	0.00
149	(E,E)-α-Farnesene	502-61-4	—	—	—	—	—	—	—	—	—	0.25±0.04	3.52	0.00
155	trans-α,α,5-Trimethyl-5-ethenyltetrahydro-2-furanmethanol	34995-77-2	0.27±0.02	0.30±0.04	0.25±0.08	0.20±0.01	0.09±0.00	0.24±0.03	0.37±0.02	0.25±0.04	0.18±0.01	0.17±0.04	2.58	0.00
159	3-Allyl-6-methoxyphenol	501-19-9	—	—	—	—	—	—	—	—	—	—	1.03	0.77

Note: FW indicates fresh weight; — indicates not detected.
